# Physical Stability and HPLC Analysis of Indian Kudzu (*Pueraria tuberosa* Linn.) Fortified Milk

**DOI:** 10.1155/2013/368248

**Published:** 2013-04-18

**Authors:** Subha Rastogi, Antariksha Katara, Madan M. Pandey, Sumit Arora, R. R. B. Singh, A. K. S. Rawat

**Affiliations:** ^1^CSIR-National Botanical Research Institute, Lucknow, India; ^2^National Dairy Research Institute, Karnal, Haryana, India

## Abstract

Functional foods provide health benefit beyond basic nutrition. Functional foods fortified with plant ingredients are well known. Ayurveda (Indian System of Medicine) has found several ways in which the medicinal benefits of herbs can be conveyed via certain foods as carriers. Milk is one such carrier which has been effectively used to deliver phytochemicals for targeted health benefits. Indian Kudzu or *Pueraria tuberosa* Linn. (Fabaceae) is an important medicinal plant of Ayurveda, and experiments suggest that it enhances the health benefits of milk when taken with milk as a carrier. Different milk combinations with *P. tuberosa* were prepared by homogenizing pasteurized toned milk with its ethanolic and hot water extracts and their stability with reference to pH and coagulation was studied over a period of 15 days. The combinations were also analyzed for puerarin, the major isoflavone C-glucoside present in *P. tuberosa*, through high-performance liquid chromatography using photo diode array detector. It was observed that there was no precipitate formation and the pH also did not change during the study period indicating their physical stability under the experimental conditions. Also there was no significant change in the content of puerarin during the study period, thereby indicating the chemical stability of the samples. These studies will be useful for developing milk nutraceuticals fortified with Indian Kudzu which has the potential to be included as an ingredient in health and functional foods.

## 1. Introduction 

The use of botanicals in foodstuffs is well established. It includes use as vegetables, fruits, herbs, and botanical food supplements. While medicinal products are intended to prevent or treat a disease or modify the way in which the body functions, food supplements and nutraceuticals are intended to complement the diet with substances possessing health-maintenance or -promoting properties [1]. Food industries have rather high demand for the products that meet the consumer's demand for a healthy lifestyle. There are many companies already capitalizing on growing consumer acceptance of food and beverages containing herbal extracts [2]. Ayurveda (Indian System of Medicine) has found several ways in which the medicinal benefits of herbs could be conveyed via certain foods as carriers. Milk is one such carrier which has been effectively used to deliver phytochemicals for targeted health benefits in the traditional Indian system of medical science. Milk is also one of the most widely consumed foods in the world and is an ideal vehicle for the fortification with these nutraceuticals.

Indian Kudzu or *Pueraria tuberosa* Linn. (Fabaceae) is an important medicinal plant of the Indian traditional system of medicine, that is, Ayurveda, and is mentioned in the Ayurvedic Pharmacopoeia of India under the name of Vidari. Powder of tubers of *P. tuberosa* (PT), commonly known as Indian Kudzu or Vidarikand in Hindi [3], is recommended for clinical use in the dose of 2–6 g/adult person [4]. It is used in traditional medicine as a fertility control agent and as an aphrodisiac, cardiotonic, diuretic and galactogogue. It has exhibited antihyperglycemic, antihyperlipidemic, antifertility in male rats, hepatoprotective, and anti-implantation activities [5–8]. It is a constituent of various formulations used as nutritive, diuretic, expectorants, and for the management of rheumatism, fever, and bronchitis [4, 9]. *P. tuberosa* tubers are rich in isoflavonoids and the important phytoconstituents are puerarin, daidzein, genistein, puetuberosanol, and tuberosin [10–13]. During the past decade, interest in these isoflavonoids has increased considerably because of the beneficial effects proposed by epidemiologists, nutritionists, and food manufacturers [14]. These isoflavonoids could interact with milk proteins, namely, bovine serum albumin [15], casein micelle [16], and *β*-lactoglobulin [17] as has been reported in case of certain food and drug preparation containing soya isoflavonoids. *In vivo* studies further revealed that these interactions often lead to reduction in lipid oxidation and improvement in antioxidant properties which are of great significance from health point of view. 

Recently, we have investigated the *in vivo* immunomodulatory and antioxidative effect of *P. tuberosa* with milk as the carrier. The results suggested that *P. tuberosa* contained active compounds that improved the therapeutic properties of milk. The milk supplemented with *P. tuberosa* extracts exhibited immunostimulatory and antioxidative properties [18]. Studies have also been carried out to determine the effect of added herb extracts on oxidative stability of ghee during accelerated oxidation condition and it was found that the ethanolic extract of vidarikand had the maximum antioxidant activity among all the herbs [19].

Since the health benefits of the nutraceuticals or functional foods containing different botanicals are due to the presence of the phytoconstituents of the added botanicals, it is important to have a biological marker and also to be able to associate that biological marker with the quality of life. Puerarin is the major constituent of *Pueraria* species. It is prescribed to treat coronary heart disease and alcoholism. It may prevent cancer, act as an antioxidant, lower serum cholesterol, and have antithrombotic and antiallergic activities [5, 20, 21]. Puerarin has exhibited antihyperglycemic effect in streptozotocin-induced diabetic rats and possesses estrogen-like biological activities. Recent studies showed that puerarin protects different cell types from damage caused by a variety of toxic stimuli. Data also suggest that puerarin might be beneficial for the treatment of Alzheimer's disease [22].

Several methods have been reported for the identification and analysis of puerarin [23]. But, to the best of our knowledge, no work on identification of *P. tuberosa* in milk nutraceuticals has been previously reported. Thus, continuing with our studies on herb-(*P. tuberosa*) milk model systems, the present work was carried out to study the stability of milk fortified with Indian Kudzu with reference to its pH and coagulation as well as to analyze Kudzu-fortified milk for puerarin, the major isoflavone C-glucoside present in *P. tuberosa*, through high-performance liquid chromatography using photo diode array detector. 

## 2. Materials and Methods

### 2.1. Chemicals and Reagents

Puerarin was obtained from Sigma-Aldrich (St. Louis, MO, USA). HPLC-grade methanol and water were obtained from Merck (Darmstadt, Germany). Whatman (Florham Park, NJ) No. 1 filter paper was used for filtration of the samples. Other chemicals and solvents were purchased from Merck Chemicals, Mumbai, India.

### 2.2. Plant Material

Tubers of *P. tuberosa* were collected from the Uttarakhand region. The sample was identified and authenticated by Dr. A. K. S. Rawat, by comparison with a reference sample preserved in the Herbarium department of NBRI. They were deposited (specimen number NBR/PH/227348) in the departmental herbal drug museum of the Pharmacognosy Division, National Botanical Research Institute, Lucknow, India, for future reference. 

### 2.3. Preparation of Ethanolic and Hot Water Extracts

The coarse air-dried, (40°–50°C), powdered tubers (500 g each) of *P. tuberosa* were extracted with ethanol by cold percolation process and with hot water by heating on a boiling water bath. The respective extracts were pooled, filtered, concentrated at reduced temperature (below 55°C) by rotary evaporation (Büchi, USA), lyophilized (Freezone 4.5; Labconco, USA) under high vacuum (133 × 104 mbar) at −40°C ± 2°C to yield the respective ethanolic (EE) and hot water (HWE) extracts, and stored at 40°C.

### 2.4. Procedure for Milk Fortification and Sterilization

Different milk combinations with *Pueraria tuberosa* were prepared by homogenizing pasteurized toned milk with extracts (EE and HWE) of *P. tuberosa*. For thermal treatment, fortified milk samples and milk controls were added into polypropylene tubes and these tubes were capped. All samples were thermally treated in an autoclave at 15 psi for 5 min. Once heating was completed, lids were tightened while still hot, and tubes were kept upright during observations and storage. Three separate batches of both the fortified milk samples and milk controls were prepared. The pH values of all combinations were determined for proper evaluation of any significant change in pH before and after storage.

### 2.5. Storage Trials, Physical Stability, and pH Tests

Fortified milk samples and milk controls were left at room temperature for 24 hr, followed by storage at 2°C to 8°C for 15 days. Stability profile was checked daily for precipitation profile and pH by bringing the samples to room temperature. Aliquots of the fortified milk samples and milk controls were observed during this period and sampled after two weeks from each of the three batches, for subsequent analyses. After sampling, the pH values of all fortified milk sample and milk controls were measured. Following the overall stability check, the analytical profiles of the 1st day and 15th day samples were compared.

### 2.6. Sample Preparation for HPLC Analysis

Fortified milk samples and milk controls were kept in freezer at lower temperature (−10°C) for 24 hours, frozen samples then freeze dried by FREEZONE 4.5 lyophilizer, and dried powder weighed to predict the yield from wet mass. Dry lyophilized powder was defatted three times with hexane (1 : 5 w/v) and finally extracted with methanol (1 : 3 w/v) by warming on water bath. Isolated fractions were then dried under reduced pressure and temperature. The dried ethanolic and hot water extracts (EE and HWE) of *P. tuberosa* as well as the dried methanolic extracts of the 1st day and 15th day samples were reconstituted in methanol and working solutions of 30 mg/mL concentrations were made. They were filtered through 0.45 *μ*m membrane filters before being subjected to HPLC analysis. 

### 2.7. HPLC Determination of Puerarin

Analyses were performed on a liquid chromatography system (Waters, Milford, MA, USA) with 515 pumps and equipped with an online degasser, a Waters Pump Control Module (PCM), an autosampler 717, a Waters 2996 photodiode array detector (PDA), and Waters Empower software. Separation was carried out using a Supelcosil LC-8-DB column (250 × 4.6 mm i.d.; 5 *μ*m pore size) with a guard column (40 × 4.6 mm i.d.) packed with the same material. The column was maintained at 25°C throughout the analysis, and detection was at 254 nm. Elution was carried out at a flow rate of 0.8 mL/min with water as solvent A and methanol as solvent B using an isocratic elution from 0–10 min with 90% of A followed by a gradient elution from 10–15 min with 90%–85% of A, 15–30 min with 85%–70% of A, 30–37 min with 70%–90% of A, and isocratic from 37 to 45 min with 90% of A. Stock solution of puerarin (1 mg/mL) was prepared in methanol and analysis was carried out under the same working conditions. Each analysis was repeated three times, and the respective retention times were averaged. Peak identification in HPLC analysis was performed by comparison of retention time with reference standard. Quantification of the compounds was achieved by use of calibration plot of the standard solution. The concentrations for the standard used for the calibration curve ranged from 1.0 *μ*g to 5.0 *μ*g for puerarin. Each run was repeated three times.

### 2.8. Validation

Validation studies were performed for determining linearity, limit of detection, limit of quantification, repeatability, and percentage recovery. Five concentration points were used to prepare the calibration curve. The calibration plot was prepared by plotting peak area against the amount of puerarin and the regression coefficient (*r* ^2^) was calculated. Limits of detection and quantification were determined by calculation of the signal-to-noise ratio. Signal-to-noise ratios of approximately 3 : 1 and 10 : 1 were used for estimating the detection limit and quantification limit, respectively. Repeatability was tested by analyzing the puerarin band after application of standard solution to the plate (*n* = 3) and calculating % RSD. Intraday as well as interday repeatability was estimated. Accuracy was determined using an added external standard. A sample of milk was spiked in triplicate with known quantities of puerarin and the percentage of recovery was calculated. The percentage of recovery rate was established from the experimental response values ((blank + standard) – blank) obtained according to the calibration curves and the real concentration of the standard added.

## 3. Results

### 3.1. Physical Stability and pH Tests

Stability profile with pH was checked daily for precipitation profile and pH by bringing the samples to room temperature. Aliquots of the fortified milk samples and milk controls were observed during this period. No precipitate formation was observed in the samples. No significant change in the pH was observed. The observations are tabulated in Table 1. 

### 3.2. HPLC Determination of Puerarin

**Figure 1:**
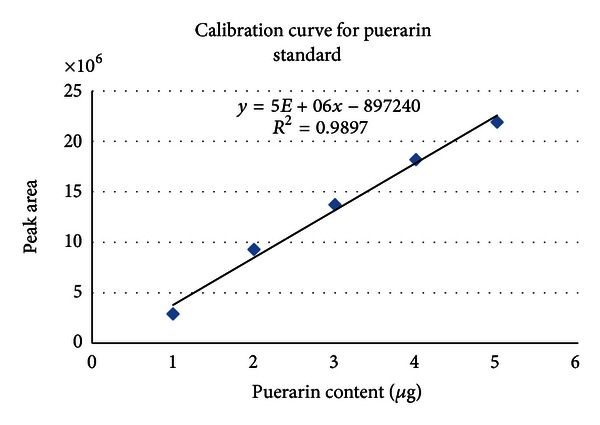
Calibration curve for standard Puerarin.

**Figure 2:**
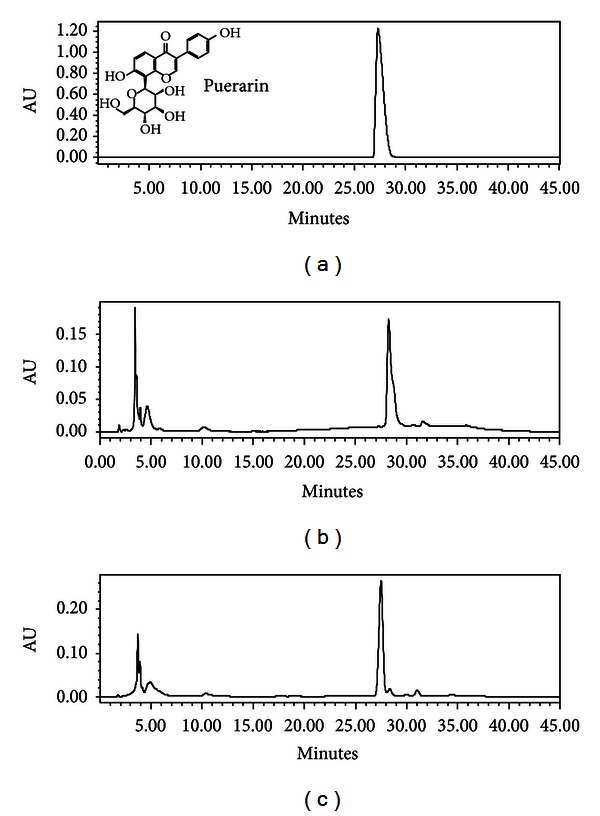
HPLC profiles of fortified milk samples (a) Puerarin standard; (b) methanolic extract of milk + 1.0% HWE of *P. tuberosa* (1st day); (c) methanolic extract of milk + 1.0% HWE of *P. tuberosa* (15th day).

**Figure 3:**
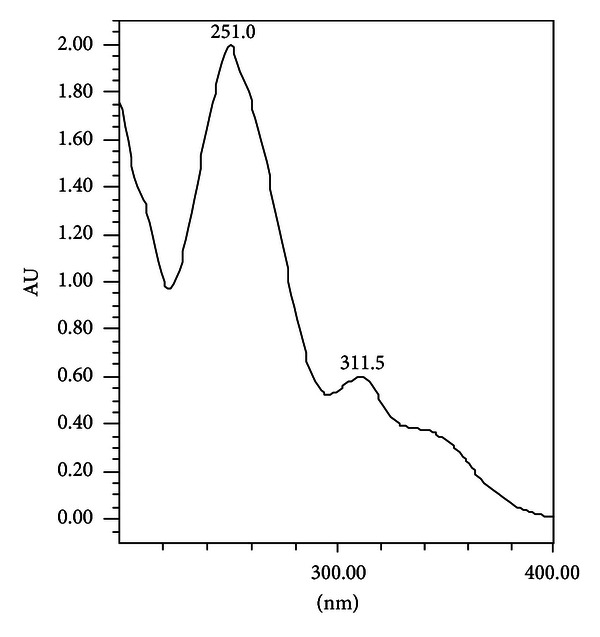
UV spectrum for Puerarin.

To determine the content of puerarin in fortified milk samples, the dried ethanolic and hot water extracts of *P. tuberosa* as well as the dried methanolic extracts of the 1st day and 15th day of fortified milk samples were reconstituted in methanol and working solutions of 30 mg/mL concentrations were made for HPLC analysis. Ten *μ*L of each sample was injected and analysis for puerarin was carried out by using HPLC under the conditions described earlier. Puerarin was monitored at 254 nm. This compound has been of interest due to its many potential health benefits. The calibration curve was plotted for puerarin (Figure 1) and the percentage content of puerarin in different samples was calculated. The ethanolic and hot water extracts of *P. tuberosa* were found to contain 5% and 1.13% puerarin, respectively. No peak corresponding to that of puerarin was observed in control milk samples.  Figure 2 shows the HPLC chromatograms of the puerarin standard and milk sample fortified with hot water extract of *P. tuberosa* as observed on 1st and 15th day, also monitored at 254 nm. The identity of Puerarin in milk samples was confirmed by comparison of the UV spectra (Figure 3) and retention time with the authentic standard and calculated as mg Puerarin per 10 mL of fortified milk. Results of the HPLC analysis are presented in Table 2.

### 3.3. Validation

Linearity, limit of detection, limit of quantification, repeatability, and percentage recovery were studied. The linear range for puerarin was 1.0–5.0 *μ*g with a correlation coefficient (*r* ^2^) of 0.989. This correlation coefficient of >0.950 was indicative of a good linear relationship between concentration and peak area in the concentration range studied. LOD and LOQ values were 300 ng and 500 ng, respectively. Both the intra- and interday R.S.D. were less than 10% over this range. At the same concentrations, accuracy ranged from 92 to 110%. The high recovery values and a high repeatability indicated a satisfactory accuracy in the method used.

## 4. Discussion

In the present investigation, different herb-milk combinations were prepared by homogenizing pasteurized toned milk with extracts (EE and HWE) of *P. tuberosa*. Storage trials for physical stability and pH monitoring of these fortified milk samples were undertaken and they were analyzed for detection and quantification of puerarin, the major isoflavone C-glucoside present in *P. tuberosa*, through high-performance liquid chromatography using photo diode array detector. A number of studies suggest that Indian Kudzu exhibits antihyperglycemic, antihyperlipidemic, hepatoprotective, antihepatotoxic, and antiimplantation activities [5–8]. Kudzu has been reported to contain high amounts of phytoestrogenic isoflavones, such as puerarin, daidzein, genistein, and their derivatives [24–26]. These compounds based on the structural similarity to internal estrogen have received much interest for the prevention of menopausal symptoms, osteoporosis, high cholesterol, heart disease, and cancer [27–32]. Also, Kudzu root powder and extract are sold in the United States, United Kingdom, and Australia as a supplement. Kudzu is often used as a single ingredient or in combination with other herbs for relieving hangover, fever, and flu; improving liver function; enhancing detoxification processes; regulating cardiac functions; and aiding weight loss [33].

Milk, being one of the most widely consumed foods in the world, is an ideal vehicle for fortification. However, it is necessary that the samples are stable and the concentration and nature of the herbs/extracts that have been added for fortification do not change on storage. It was observed that there was no precipitate formation and the pH also did not change during the study period. This indicated that the milk samples were physically stable under the experimental conditions mentioned above. Also, the biomarker used for chemical analysis was puerarin, which is the major constituent of Kudzu. HPLC analysis results showed that the puerarin content can directly be a measure of the amount of ethanolic or hot water extracts of *P. tuberosa* added for fortification. Also there was no significant change in the content of puerarin during the study period, thereby indicating the chemical stability of the samples. These studies will be useful for developing milk nutraceuticals fortified with Indian Kudzu which has the potential to be included as an ingredient in health and functional foods. 

## 5. Conclusions

A modern lifestyle is fast paced and mostly hurried where most people battle with time poverty. As a result, it is often difficult to find the time and energy to eat correctly and supply your body with the correct type of nutrition. On top of that, an individual's health and nutrition needs do also change throughout his or her life. It is for this reason that development of functional foods and nutraceuticals with special health-promoting benefits is the need of the day. Herbal extracts in all their forms possess arguably the greatest potential for innovative functional food products. There are many companies already capitalizing on growing consumer acceptance of food and beverages containing herbal extracts [2] although the use of these extracts in milk and milk products is quite a recent development. Milk is also one of the most widely consumed foods in the world and is an ideal vehicle for fortification with these nutraceuticals. Also, numerous nutraceutical combinations have entered the international market through exploration of ethnopharmacological claims made by different traditional practices. These foods or nutraceuticals construct a health-promoting, disease-preventing diet with protective substances. The rich nutrient food intake will provide maximum protection against not only infections, asthma, and allergies, but also against heart disease and cancer in adulthood. However, before the full market potential can be realized, the consumers need to be assured of the safety and efficacy of functional foods. Future research will focus on mechanisms by which food components such as phytochemicals positively affect health, and whether these components work independently or synergistically. 

## Figures and Tables

**Table 1:** Stability profile of milk samples.

Milk samples	Day 1 sample		Day 5 sample		Day 10 sample		Day 15 sample	
	Precipitate	pH	Precipitate	pH	Precipitate	pH	Precipitate	pH
Milk + 0.5% EE (w/v)	Absent	6.50	Absent	6.53	Absent	6.55	Absent	6.58
Milk + 1.0% EE (w/v)	Absent	6.46	Absent	6.59	Absent	6.60	Absent	6.69
Milk + 0.4% HWE (w/v)	Absent	6.42	Absent	6.50	Absent	6.59	Absent	6.63
Milk + 0.7% HWE (w/v)	Absent	6.43	Absent	6.45	Absent	6.48	Absent	6.51
Milk + 1.0% HWE (w/v)	Absent	6.42	Absent	6.50	Absent	6.55	Absent	6.59
Milk + 0.01% puerarin (w/v)	Absent	6.52	Absent	6.60	Absent	6.64	Absent	6.68
Milk (control)	Absent	6.61	Absent	6.65	Absent	6.68	Absent	6.71

EE: ethanolic extract of *P. tuberosa.*

HWE: hot water extract of *P. tuberosa.*

**Table 2:** Puerarin content in Indian Kudzu fortified milk samples.

Milk sample	Day 1 sample	Day 15 sample
	(mg/10 mL fortified milk)	(mg/10 mL fortified milk)
Milk + 0.5% EE (w/v)	2.39 ± 0.03	2.34 ± 0.03
Milk + 1.0% EE (w/v)	5.25 ± 0.04	5.19 ± 0.16
Milk + 0.4% HWE (w/v)	0.41 ± 0.02	0.34 ± 0.04
Milk + 0.7% HWE (w/v)	0.81 ± 0.03	0.81 ± 0.03
Milk + 1.0% HWE (w/v)	1.24 ± 0.05	1.27 ± 0.02
Milk + 0.01% puerarin (w/v)	1.03 ± 0.09	0.97 ± 0.02

EE: ethanolic extract of *P. tuberosa.*

HWE: hot water extract of *P. tuberosa.*
